# Antimicrobial peptides derived from human ameloblastin targeting biofilms

**DOI:** 10.1186/s12903-025-07433-w

**Published:** 2025-12-02

**Authors:** Veronika Vetyskova, Petra Kasparova, Lucie Bednarova, Miroslav Hajek, Jan Luxa, Alejandro Barrantes Bautista, Jane Elin Reseland, Olga Matatkova, Jan Masak, Jiri Vondrasek, Kristyna Vydra Bousova

**Affiliations:** 1 Institute of Organic Chemistry and Biochemistry of the Czech Academy of Sciences, Flemingovo namesti 2, Prague, 16000 Czechia; 2 Department of Biotechnology, University of Chemistry and Technology Prague, Technicka 5, Prague, 166 28 Czechia; 3 Department of inorganic chemistry, University of Chemistry and Technology, Technická 5, Prague, Czechia; 4 Oral Research Laboratory, Institute of Clinical Dentistry, University of Oslo, Oslo, Norway; 5 Department of Biomaterials, Institute of Clinical Dentistry, University of Oslo, Oslo, Norway

**Keywords:** Ameloblastin, Antimicrobial peptides, Bacterial biofilm, Tooth enamel, IDP

## Abstract

Oral biofilm-related diseases, such as dental caries and periodontitis, remain among the most prevalent global health issues and are increasingly complicated by antibiotic resistance and biofilm persistence, which limit the effectiveness of conventional treatments. This study addresses the challenges by exploring antimicrobial peptides (AMPs) derived from ameloblastin (AMBN), a protein integral to dental biomineralization and categorized as an intrinsically disordered protein. In humans, the AMBN gene encodes two isoforms, ISO I and ISO II, with distinct but not fully understood functions. Four AMBN ISO I–derived peptides (A, Am, B, Bm) were designed, synthesized, and tested for antimicrobial and antibiofilm activity. Peptides A and Am moderately inhibited biofilms of *E. faecalis*, *S. aureus*, and *E. coli* (MBIC₅₀ within 50–300 µM), including resistant isolates, while B and Bm were more effective against Gram-positive strains, showing the strongest effect against methicillin-resistant *S. aureus* CNCTC 6271. Cytotoxicity assays showed > 90% cell viability at 50 µM and IC₅₀ >100 µM for HCT116 and > 300 µM for HUVEC cells, with haemolytic activity > 300 µM. Stable immobilization of peptides on titanium surfaces was confirmed by XPS and QCM-D techniques, supporting their potential as low-toxicity antimicrobial coatings for medical implants.

**Supplementary Information:**

The online version contains supplementary material available at 10.1186/s12903-025-07433-w.

## Background

Ameloblastin (AMBN), also known as amelin or sheatlin, is an intrinsically disordered protein essential for enamel matrix formation during amelogenesis [1, 2]. The enamel matrix formation comprises several key proteins, including amelogenin, enamelin, tuftelin and AMBN [1, 3, 4], which are sequentially processed by proteases such as kallikrein 4 (KLK-4) and enamelysin (matrix metalloproteinase-20, MMP-20). In addition to their structural roles, peptides derived from enamel matrix proteins have demonstrated antimicrobial properties [5, 6] suggesting a dual function in enamel development and microbial defence. In our previous work it was identified proteolytic profiles of recombinant human AMBN (hAMBN) [7, 8], revealing peptides that were tested for potential antimicrobial activity against bacterial biofilms in this work.

Antimicrobial peptides (AMPs) provide alternatives to traditional antibiotics, especially in addressing biofilm-associated infections that are often resistant to conventional antibiotic treatments [9–11]. AMPs can commonly act as a disruptor of bacterial membranes, inhibitor biofilm formation, suppressor motility-related genes, and inhibitor DNA, RNA, and protein synthesis [12]. AMPs were found to be effective against a wide range of species, including bacteria, fungi, viruses, and parasites [13, 14]. Given the reduced sensitivity of biofilm-associated bacteria to antibiotics and rising antibiotic resistance, novel therapeutic approaches are critical. Specifically biofilm-related infections, like those in cystic fibrosis or wound infections, exhibit high treatment failure rates [14, 15]. Bacteria within biofilms exhibit significantly enhanced tolerance to antimicrobial agents and host immune defenses due to their extracellular polymeric matrix, metabolic diversity, and coordinated signalling networks [16]. This resilience contributes to the chronicity of oral and implant-associated infections and reduces the efficacy of conventional antibiotic therapies. The increasing prevalence of multidrug-resistant pathogens further highlights the need for alternative antimicrobial strategies [17]. Recent studies have further demonstrated AMPs multifunctional roles in oral health, influencing bacterial adhesion, biofilm architecture, and immune regulation [9, 18]. Comprehensive reviews have also emphasized the mechanistic diversity and clinical translation potential of AMP [19–21], underscoring the importance of optimizing their stability, selectivity, and toxicity profiles for biomedical and oral health applications [22–24].

The antibiofilm activity of AMPs are evaluated using metrics such as the minimum biofilm inhibitory concentration (MBIC) and minimum biofilm eradication concentration (MBEC). Importantly, AMPs exhibiting antimicrobial activities have to be also evaluated for their low toxicity toward mammal cells [25–27], with this feature they can represent suitable tool for broad range of therapeutic applications [28]. Furthermore, AMPs may display synergy with classical antibiotics, neutralize endotoxins, and show high activity in animal models [29, 30]. The other advantages of AMPs can be that their resistance is rare due to the affinity for bacterial membranes and rapid action [31]. AMPs can also stimulate the immune response, enhancing the host’s ability to combat infections [15, 32]. One of the most promising application of AMPs exhibiting MBIC or MBEC functions is their immobilization on medical implants (e.g. TiO_2_) to prevent biofilm formation, addressing issues with systematic antibiotics transport and toxicity due to the treatment [33–36].

Our previous work described the identification of proteolytic profiles of hAMBN by mass spectrometry analysis in order to specify cleavage sites by MMP-20 and KLK-4 proteases as they are specific for enamel environment [37, 38]. The recombinant hAMBN was expressed in an *E. coli* system, identifying peptides formed by the cleavage of full-length hAMBN [7]. In this study, it was compared the hAMBN ISO I identified peptides with predictions for antimicrobial activities from bioinformatics tools. Four hAMBN peptides were identified, engineered and tested overall for antibacterial efficacy in solution and against biofilms. The tested bacteria forming biofilms (*Enterococcus faecalis*,* Staphylococcus aureus*, and *Escherichia coli*) were selected based on their natural environment close to hAMBN expression in human body which is oral cavity [39–41]. Beside the MBIC and MBEC activities of the peptides, they also exhibited minimal haemolytic and cytotoxic activities, highlighting their therapeutic potential. This research can underscore AMBN’s multifunctionality in enamel development and its potential as an antimicrobial agent, offering promising implications for oral therapeutic and implant-related applications.

## Methods

### Design of antimicrobial peptides

Bioinformatic tools, including AmpGram [42], AmPEP [43], AntiMPmod [44], and DBAASP [45] were used to predict AMPs within the AMBN ISO I sequence. Cleavage profiles of AMBN ISO I by MMP-20 and KLK-4, determined in our previous studies, were integrated with these predictions to select candidate peptides. Structural modeling using PEP-FOLD [46] and visualization in BIOVIA Discovery Studio [47] were employed to evaluate the peptides’ secondary structure and potential antimicrobial properties.

### Peptides synthesis

The anticipated peptides derived from AMBN (UniProtKB - Q9NP70), specifically A, Am, B and Bm (sequences: A [QGSTIFQIARLISHGPM], Am [QGHTIFQIARLISHGPM], B [STIFQIARLISHGPMPQNKQSP], Bm [STIFQIARLISHG**A**MAQNKQSP**G**]) were synthesized using solid-phase peptide synthesis with the standard N-Fmoc protocol. The synthesis was carried out on TentaGel S RAM resin (431 mg with 0.24 mmol/g substitution) using a PS3 automated peptide synthesizer (Protein Technologies, Tucson, AZ). N-Fmoc-protected amino acids (10 eq.) were coupled with a 0.4 M N-methylmorpholine solution in N, N-dimethylformamide (DMF, 20 eq.) and HBTU (10 eq.) in N-methyl-2-pyrrolidone. The α-amino groups’ protective groups were removed with a 20% piperidine solution in DMF. Peptides were fully deprotected and cleaved from the resin with a TFA/H2O/triisopropylsilane mixture (92.5:5:2.5) for 2 h, followed by precipitation in tert-butyl methyl ether. Purification of the peptides was achieved using reverse-phase HPLC (Jasco Inc., Easton, MD, USA) on a YMC-Pack ODS-AM column (5 μm, 250 × 20 mm). The identity and purity of the synthetic peptides were confirmed using an Agilent 1260 HPLC coupled with an Agilent 6530 ESI-TOF and Agilent Jet Stream technology, ensuring a purity greater than 97%.

### Microorganisms and cultivation conditions

1. *Enterococcus faecalis* DBM 3075, a clinical isolate, was obtained from Motol University Hospital in Prague, Czech Republic, and provided by the Department of Biochemistry and Microbiology at the University of Chemistry and Technology, Prague. Control strains *Enterococcus faecalis* CNCTC 5530 (ATCC 51299) and *Enterococcus faecalis* CNCTC 5483 (ATCC 29212) were sourced from the Czech Collection of Type Cultures of Microorganisms in Prague. Additionally, *Enterococcus faecalis* M-1, another clinical isolate, was also obtained from Motol University Hospital.
2. *Staphylococcus aureus* CNCTC 5670 (ATCC 12600, type strain) and *Staphylococcus aureus* CNCTC 6271 (ATCC 43300, methicillin-resistant strain) were sourced from the Czech National Collection of Type Cultures of Microorganisms in Prague. *Staphylococcus aureus* DBM 3178 (ATCC 29213) was provided by the Department of Biochemistry and Microbiology at the University of Chemistry and Technology, Prague. *Staphylococcus aureus* M-1, methicillin-resistant strain isolated from a patient with an artificial knee implant infection, was procured from Motol University Hospital.
3. *Escherichia coli* DBM 3125 (ATCC 10536) and *Escherichia coli* DBM 3138 (ATCC 8739) were kindly provided by the Department of Biochemistry and Microbiology at the University of Chemistry and Technology, Prague. Escherichia coli CCM 4787 (O157) and *Escherichia coli* CCM 7372 (strain B) were sourced from the Czech Collection of Microorganisms in Brno, Czech Republic.

*Enterococcus faecalis* and *Staphylococcus aureus* were cultured in tryptone soya broth (TSB) medium, while *Escherichia coli* was cultured in Luria-Bertani (LB) broth. All cultures were grown at 37 °C with shaking at 150 rpm for 24 h for further experimental procedures.

### Effect of AMPs on suspension growth of microorganisms

The impact of antimicrobial peptides (AMPs) on the growth of microorganisms in suspension was evaluated using a Bioscreen C microculture device (LabSystems, Finland) [48]. Precultures were initially adjusted to an optical density (OD) of 0.1 at 600 nm (CFU/ml = 2.5 × 10^7^). A volume of 30 µl of the prepared inoculum was added to a polystyrene microtiter plate (Honeycomb 2, Growth Curves, USA) along with 290 µl of growth media. The cultures were incubated in the microcultivation device at 37 °C and 150 rpm for 24 h, with OD measurements taken every 30 min. Growth curves were generated to analyse cell growth in the suspension for each well, with control samples lacking AMPs (MQ water, Sigma Aldrich, MA, USA). Each condition was tested in triplicate. The minimum inhibitory concentration (MIC_50_) was defined as the lowest concentration of the antimicrobial agent that resulted in a 50% reduction in microorganism growth after 24 h, compared to growth without the antimicrobial agent. The AMPs were diluted in MQ water (Sigma Aldrich, MA, USA) ranging from 1 to 1000 µM concentrations.

### Effect of AMPs on biofilm formation of microorganisms

The impact of antimicrobial peptides (AMPs) on biofilm formation was investigated using a protocol adapted from a previously published method [49]. The inoculum, cultured to an OD_600nm_ of 0.8 (CFU/ml = 2 × 10^8^), was added to a microtiter plate (TPP, Trasadingen, Switzerland) in 210 µl aliquots. Then, 70 µl of growth media and the AMPs were added and mixed (AMPs diluted in MQ water in 1–1000 µM concertation range). Biofilms were allowed to form under static conditions at 37 °C for 24 h. Control samples (MQ water, Sigma Aldrich, MA, USA) without AMPs were included in each experiment. All experiments were performed in triplicate to ensure accuracy and reproducibility.

### Effect of AMPs on the eradication of already mature biofilms from microorganisms

The investigation into the impact of AMPs on the eradication of mature biofilms followed a similar procedure to the one described previously. Initially, biofilms were cultured without antimicrobial peptides. The inoculum was grown to an OD_600nm_ of 0.6 (CFU/ml = 1.5 × 10^8^) and then added to a microtiter plate (TPP, Switzerland) in 200 µl volumes. Biofilms were allowed to form under static conditions at 37 °C for 24 h. After this initial incubation, the biofilms were washed with a sterile saline. Solutions containing the peptides and growth medium were then added to the wells in a final volume of 200 µl. The biofilms were incubated again under static conditions at 37 °C for 24 h. As in the previous experiments, control samples without AMPs were included. Each experiment was conducted in triplicate to ensure reliability and reproducibility. The metabolic activity of bacterial biofilms was assessed using resazurin as an indicator and measuring the increase in resorufin absorbance as described in 2.7.

### Determination of the metabolic activity of bacterial biofilms

The metabolic activity of biofilm cells was assessed using resazurin viability assay (Thermo Fisher Scientific, MA, USA) [50]. After washing the biofilm, 25 µl of D-glucose solution (180 g/l in saline), 25 µl of resazurin solution (0.15 g/l in physiological solution), and 100 µl of physiological solution were added. For *E. coli* biofilms, measurements were taken either immediately or after a 30-minute incubation at 37 °C depending on colour change between untreated samples and blank wells. Fluorescence intensity was measured at 545/575 nm using an Infinite M200 Pro Reader plate spectrophotometer (Tecan, Männedorf, Switzerland). Background fluorescence was subtracted from the data, and the mean and standard deviation were calculated and converted to relative percentages for comparison of strains and peptides. All experiments were conducted independently three times.

The minimum biofilm inhibitory concentrations (MBIC_50_ and MBIC_80_) were determined as the lowest concentration of an antimicrobial agent that reduced the metabolic activity of biofilm cells by 50% or 80% compared to the control (cells without any antimicrobial agent) after 24 h of cultivation. The minimum biofilm eradication concentration (MBEC_50_ and MBEC_80_) was identified as the lowest concentration of an antimicrobial agent that reduced the metabolic activity of cells in mature biofilms by 50% or 80% compared to the control.

### Haemolytic activity assay

For haemolytic activity assay was collected fresh human blood from one of the authors (MH) on the day of the experiment. Written informed consent was obtained prior to collection, and all procedures complied with institutional ethical standards (IOCB CAS, Czech Republic). A volume of 1 ml of whole blood diluted with 9 ml of phosphate-buffered saline (PBS) and centrifuged at 800 g for 15 min at room temperature. The resulting red blood cell pellet was washed five times with 10 ml of PBS, with centrifugation at 800 g for 15 min at room temperature after each wash. The supernatant was discarded after each wash, leaving the washed red blood cell pellet. The pellet was then diluted with PBS to a final concentration of 0.5% (v/v). A volume of 50 µl of this final suspension was transferred to a 96-well microtiter plate. Subsequently, 50 µl of double-concentrated peptide solutions (containing the tested antimicrobial peptides at concentrations ranging from 12.5 to 300 µM, and Melittin (synthetised at IOCB CAS, Czech Republic; served as positive control) at 0.024 to 50 µM in twofold dilutions), PBS, and 0.5% Triton X-100 were added to the wells. The plate was incubated for 60 min at 37 °C, followed by centrifugation at 1000 g for 15 min at room temperature. Then, 40 µl of the supernatant from each well was transferred to a transparent 384-well plate. The absorbance of the supernatant was measured at 415 nm using a Cytation 3 microplate reader (BioTek, USA).

### Cytotoxicity assay

Two types of cell cultures were employed for the cytotoxicity assay: primary human umbilical vein endothelial cells (HUVEC) from pooled donors (Lonza, Basel, Switzerland) and human colon carcinoma cell line HCT 116 (ATCC). The HCT116 human epithelial cell line was selected as a sensitive model system for preliminary cytotoxicity screening of antimicrobial peptides. Its use is well established for evaluating general peptide-induced membrane effects prior to testing on specialized oral cell models. HUVEC cells were cultured in the kit containing EBM^TM^−2 Basal Medium (CC-3156) and EGM^TM^−2 SingleQuots^™^ Supplements (Lonza, Basel, Switzerland) while HCT 116 cells were cultured in McCoy’s 5 A growth medium (Sigma Aldrich) supplemented with 10% (v/v) heat-inactivated fetal bovine serum (FBS). The cultures were maintained at 37 °C in a humidified atmosphere with 5% CO2. When cells reached 80–90% confluence, medium change was performed twice a week using a solution of 0.25% trypsin and 0.53 mM EDTA. Both cell cultures were maintained under similar conditions, ensuring consistent and optimal growth environments for accurate cytotoxicity assessments. The cytotoxicity of the tested antimicrobial peptides (AMPs) was assessed using the CellTiter-Glo^®^ Luminescent Cell Viability Assay kit (Promega, Madison, WI, USA). Cells were cultured as described previously, and the assay was performed following the manufacturer’s instructions. Specifically, 10,000 HUVEC cells (50 µl) were seeded into each well of a white 96-well plate (BD Biosciences™, San Jose, CA, USA). After a 24-hour incubation period, 50 µl of 600 µM peptide solutions or EGMTM-2 medium (as a control) were added to each well.

Following an additional 72-hour incubation, 100 µl of CellTiter-Glo^®^ reagent was added to each well. The plate was mixed for 2 min at 400 rpm on an orbital shaker in the dark, and the luminescence signal was allowed to stabilize at room temperature for 10 min. Luminescence, which directly correlates with the number of viable cells, was measured using a Cytation 3 microplate luminometer (BioTek, USA).The resulting data were normalized, and IC50 values were calculated through non-linear regression analysis, assuming a sigmoidal concentration response curve with a variable Hill slope, using GraphPad PRISM^®^ 7 software (San Diego, CA, USA, www.graphpad.com).

### Circular dichroism spectroscopy of AMPs

Circular dichroism (CD) spectra were obtained using a Jasco-1500 spectropolarimeter (JASCO Inc., Easton, MD, USA) at room temperature. The spectra were recorded over a wavelength range of 190 to 300 nm using a 0.1 cm cylindrical quartz cell. The instrument was set to standard sensitivity, with a 1 nm bandwidth, a scanning speed of 10 nm/min, and a response time of 8 s. Peptides were also measured in deionized water (pH 7.5) and with the addition of trifluoroethanol (TFE) at concentrations of 0%, 25%, and 50% (v/v). The sample concentration kept constant (0.1 mg/ml) for all experiments. After baseline correction, the spectra were expressed as molar ellipticity θ (deg · cm² · dmol⁻¹) per residue. The numerical analysis of secondary structures was performed using the CD Pro software package using CONTIN program [51].

### Confocal fluorescence spectroscopy

The localization of peptide Fl-LL-III/43 in adhered cells was visualized using spinning disk confocal microscopy (SDCM; Olympus/Andor, Japan/Ireland) according to a modified method of Slaninová et al. 2011. The adhered *C. albicans* ATCC MYA-2876 cells in 96-well microtiter plates were prepared in the same way as described above (for 2 h, in the absence of Fl-LL-III/43), the difference being in the use of plates with thin polystyrene bottom suitable for microscopy (Greiner Bio-One, Frickenhausen, Germany). Fl-LL-III/43 (2 µM) was added to the adhered cells and the location of the peptide was visualized during 5.5 min of incubation in darkness at room temperature. Double staining of Fl-LL-III/43 (488 nm) with DAPI (405 nm; Sigma-Aldrich, Czech Republic) was used to distinguish the location of Fl-LL-III/43 in *C. albicans* cells.

### Coating on titanium layer via polydopamine layer

Grinded and polished disc-shaped titanium test implants (for dental purpose) were kindly provided by Department of Biomaterials, Institute of Clinical Dentistry, University of Oslo, Oslo, Norway. Titanium discs with a diameter of 6.25 mm and a height of 1.95 mm were blasted on one side with TiO_2_ powders of grain size 180–220 μm [52]. The discs were mounted on a silicon holder and positioned approximately 20 mm from the jet nozzle. The TiO_2_ particle stream impacted the surface at a 90° angle with an air pressure of 0.4 MPa. Each implant was blasted with repeated horizontal and vertical movements for a duration of 8 s. Excess TiO_2_ was removed using an air jet nozzle. After blasting, the discs were cleaned in an ultrasonic bath with 40 vol% NaOH followed by 50 vol% HNO_3_ for 30 min to remove contaminants. The discs were then rinsed with 100% ethanol in an ultrasonic bath for three cycles of 10 min each. After rinsing with deionized water, the discs were stored in water. For further preparation, the discs were immersed in a dopamine hydrochloride solution (2 mg/ml) prepared in Tris-Base buffer (10 mM, pH 7.4) and 100 mM NaCl for about 12 h in darkness [53, 54]. Then the discs were carefully washed in MQ water for 3 times and air dried. 300 µM solution of A and Am peptides were prepared by dissolving in buffer containing 10 mM Tris-base (pH 7.4) and 100 mM NaCl buffer. Each disc was placed into a cavity and treated with 10 µl of the peptide (C = 100 μm) and incubated for 24 h in 37 °C.

### X-ray photoelectron spectroscopy

Solutions of antimicrobial peptides (AMPs) at a concentration of 300 µM were prepared using a buffer containing 10 mM Tris-HCl (pH 7.5) and 100 mM NaCl. Each disc was placed into a cavity of a 24-well microtiter plate and treated with 10 µl of the AMP solution [35]. A control sample without the peptide was also included. The discs were then air-dried and analyzed by X-ray photoelectron microscopy (XPS) in order to confirm specific coating of AMPs on the TiO^2^ surface. The XPS was performed using the ESCAProbeP from Omicron Nanotechnology Ltd. (London, England), with exposed area dimensions of 1 × 13 mm2 that were analyzed. The X-ray source was monochromatized to 1486.7 eV, and each measurement was performed with a step of 0.1 eV. CasaXPS, ver. 2.3.24 software was used for spectral evaluations.

### Quartz crystal microbalance with dissipation

Peptide adherence to Ti sensors (with and without DOPA nanocoatings) was monitored in quartz crystal microbalance with dissipation (QCM-D) QSense^®^ window module (QFM401) using a QCM-D QSense^®^ E4 (Biolin Scientific). TiO_2_ sensors (QSX 310, Biolin Scientific) were used and cleaned according to the manufacturer’s protocol before and after each experiment. The procedure includes sonication in 2% SDS, washing with MQ water and EtOH, and final UV-ozone treatment. The QCM-D chambers were cleaned with 2% SDS for 10 min and extensively flushed with water (> 15 min) prior to the measurement. After the equilibration of the sensors in buffer (10 mM Tris-HCl supplemented with 100mM NaCl, pH 7.5) at 21 °C, the A and Am peptides were pumped into the flow chamber at a flow rate of 0.1 ml/min, and after 2 min the flow rate was reduced to 0.01 ml/min. The peptides were left to adsorb for 40 min, and then the sensors were rinsed with peptide free buffer at a flow rate of 0.1 ml/min.

## Results

### Antimicrobial peptides design

**Fig. 1:**
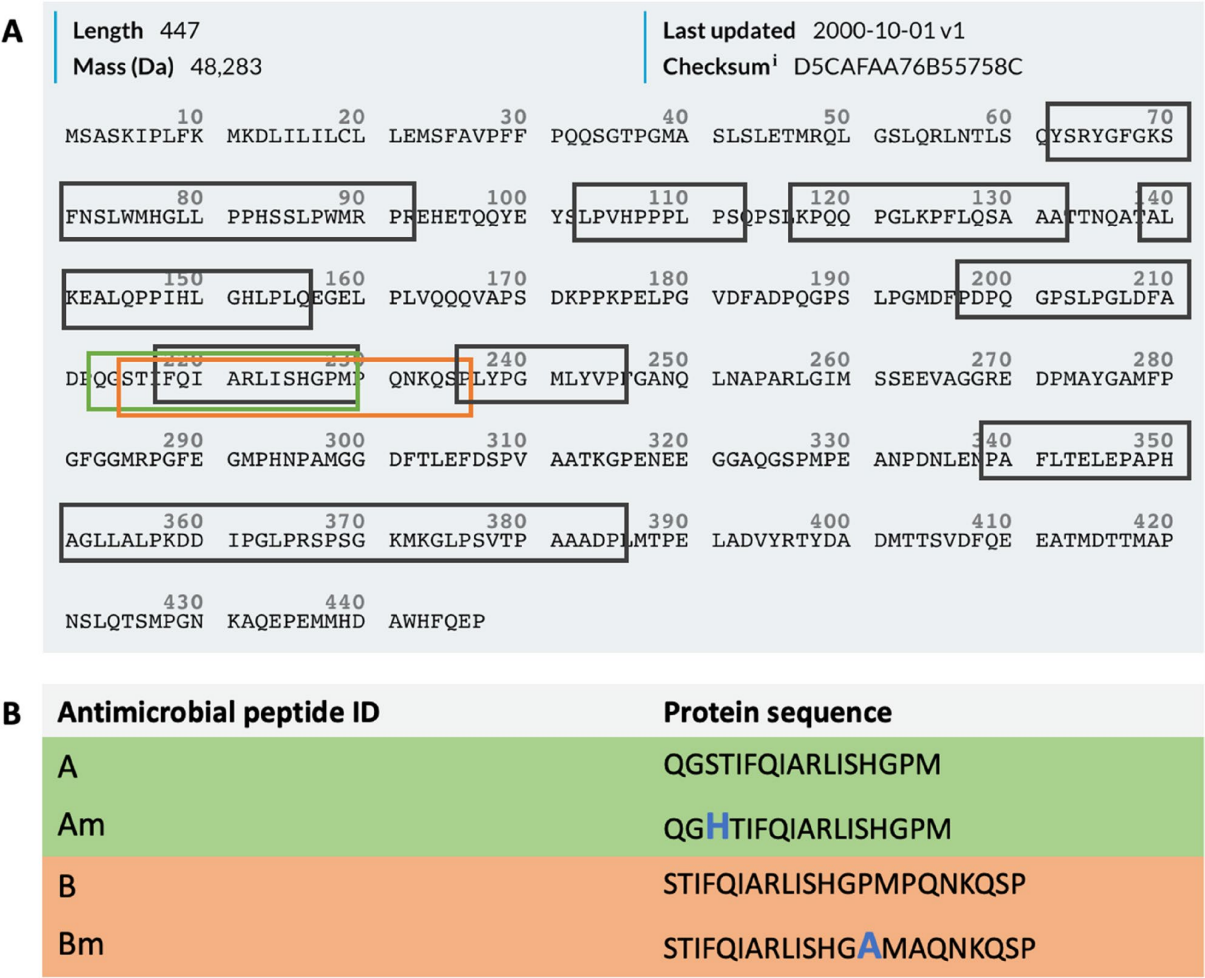
Antimicrobial peptides identification and design. (**A**) The analysis of AMBN ISO I for potential antimicrobial activity of predicted peptides. The analysis was conducted using AmpGram bioinformatics tool [7] (highlighted in grey). Final predicted and designed AMPs are labelled in orange and green boxes [42]. (**B**) Identified and partially modified potential antimicrobial peptides A and B with their engineered versions Am and Bm. Blue amino acids stands for mutations in the peptide

For the design of antimicrobial peptides (AMPs), bioinformatics tools were employed, including AmpGram [28], AmPEP [29], AntiMPmod [30], and DBAASP [31], to identify potential antimicrobial sequences from AMBN ISO I. The PEP-FOLD tool [32] was used to predict the structures of the identified peptides, while BIOVIA Discovery Studio [33] was utilized for peptide visualization. The visual representation, Fig. 1 A shows the result of AMBN ISO I proteolytic cleavage peptides (grey boxes) identified as described previously [7] with peptides in the AMBN ISO I sequence found with a probability of an antimicrobial activity greater than 50% as predicted by the AmpGram tool [28]. The sequences predicted by the bioinformatics tools were compared with data obtained through the proteolysis of AMBN ISO I using MMP-20, KLK-4 [7]. Two of the AMPs were derived directly from the AMBN sequence, identified as A and B. The A and B peptides were further engineered using molecular modeling techniques to enhance their alpha-helical content and to improve the solubility, resulting in the engineering of new Am and Bm peptides (Fig. 1B). PEP-FOLD [32], a structure prediction tool, was employed to predict the structures of A and B, which were then compared with the predicted structures of Am and Bm, confirming increased alpha-helical content. The theoretical antimicrobial activity of the peptides Am and Bm was reassessed using the DBAASP bioinformatics tool [31]. Initially, the lengths of the peptides were extended, and these elongated peptides were then ranked based on their predicted antimicrobial activity using DBAASP [31].

### Antimicrobial activity of antimicrobial peptides against bacterial biofilms

**Fig. 2:**
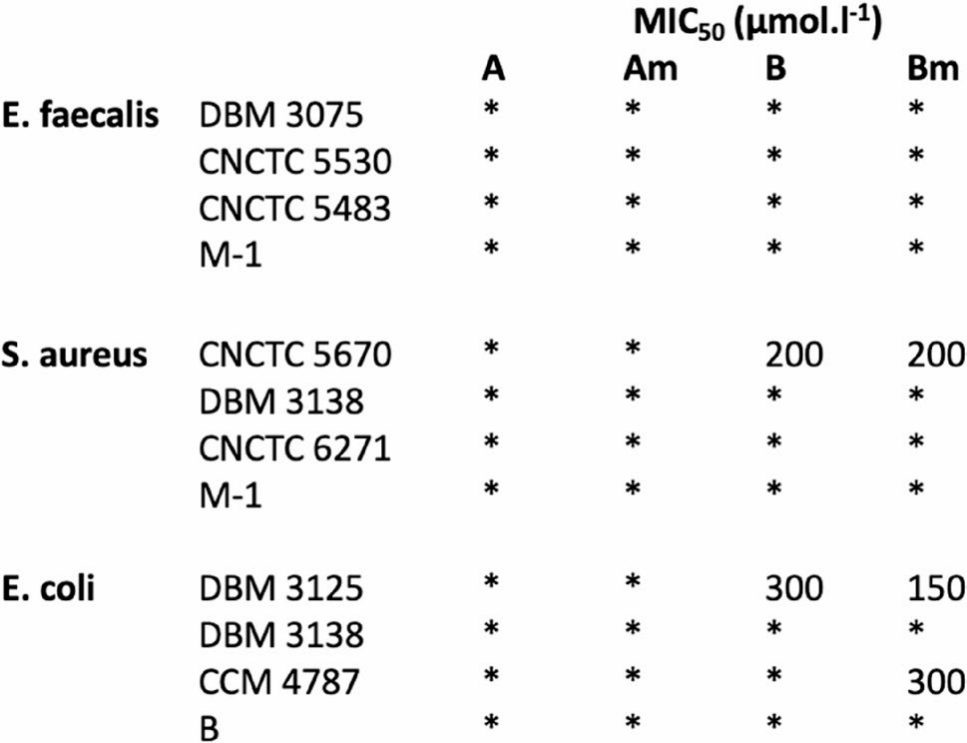
AMPs antimicrobial activity analysis. The effect of studied antimicrobial peptides A, Am, B, Bm peptides on the suspension growth of *Enterococcus faecalis*,* Staphylococcus aureus* and *Escherichia coli* referred as MIC_50_. * Not detected in the concentration range (50–300 µM)

**Fig. 3:**
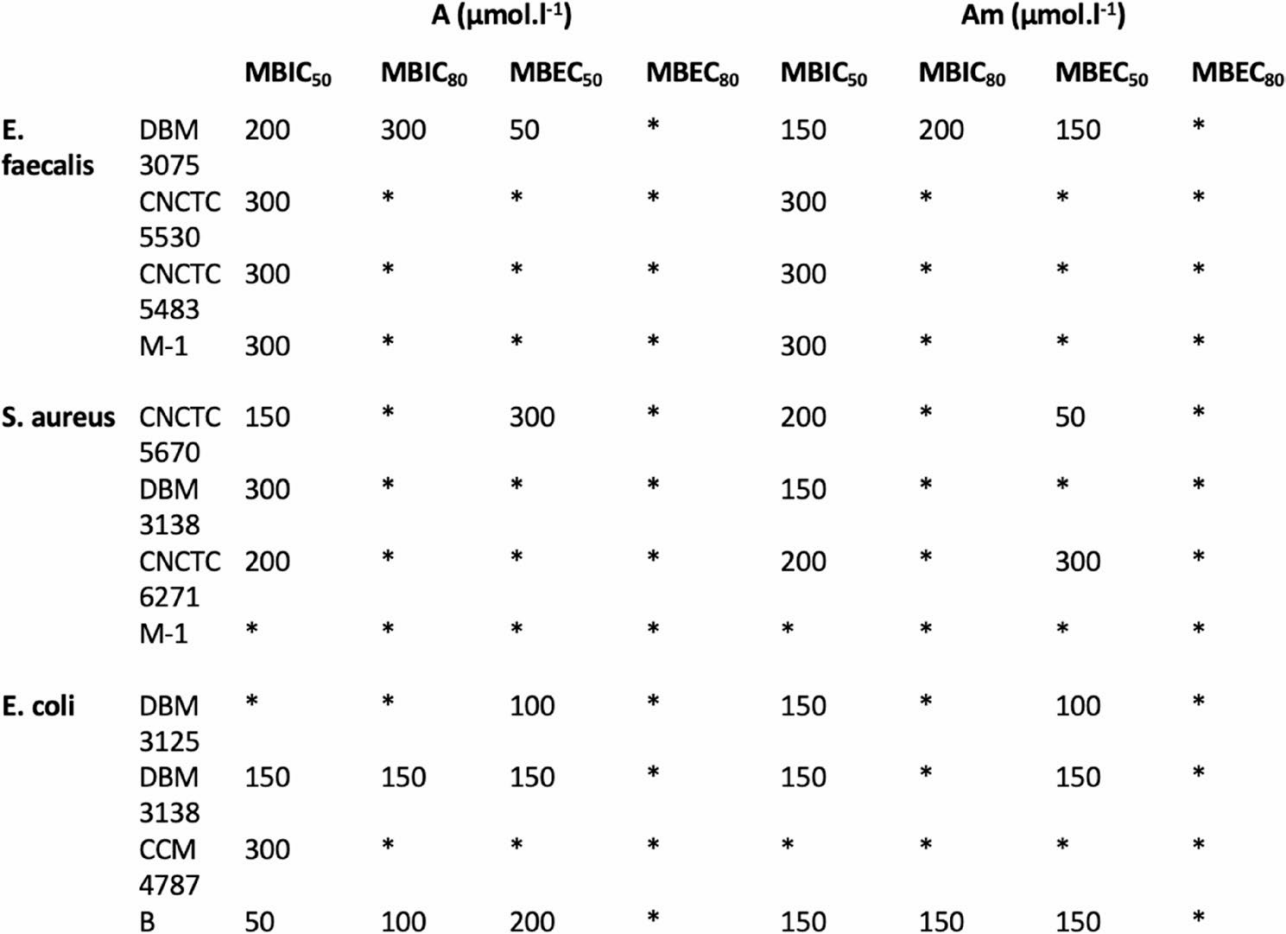
AMPs antimicrobial activity analysis. The effect of A and Am peptides on biofilm formation and eradication of mature biofilm of Enterococcus faecalis, Staphylococcus aureus and Escherichia coli referred as MBIC50, MBIC80, MBEC50 a MBEC80. * Not detected in the concentration range (50–300 µM)

**Fig. 4:**
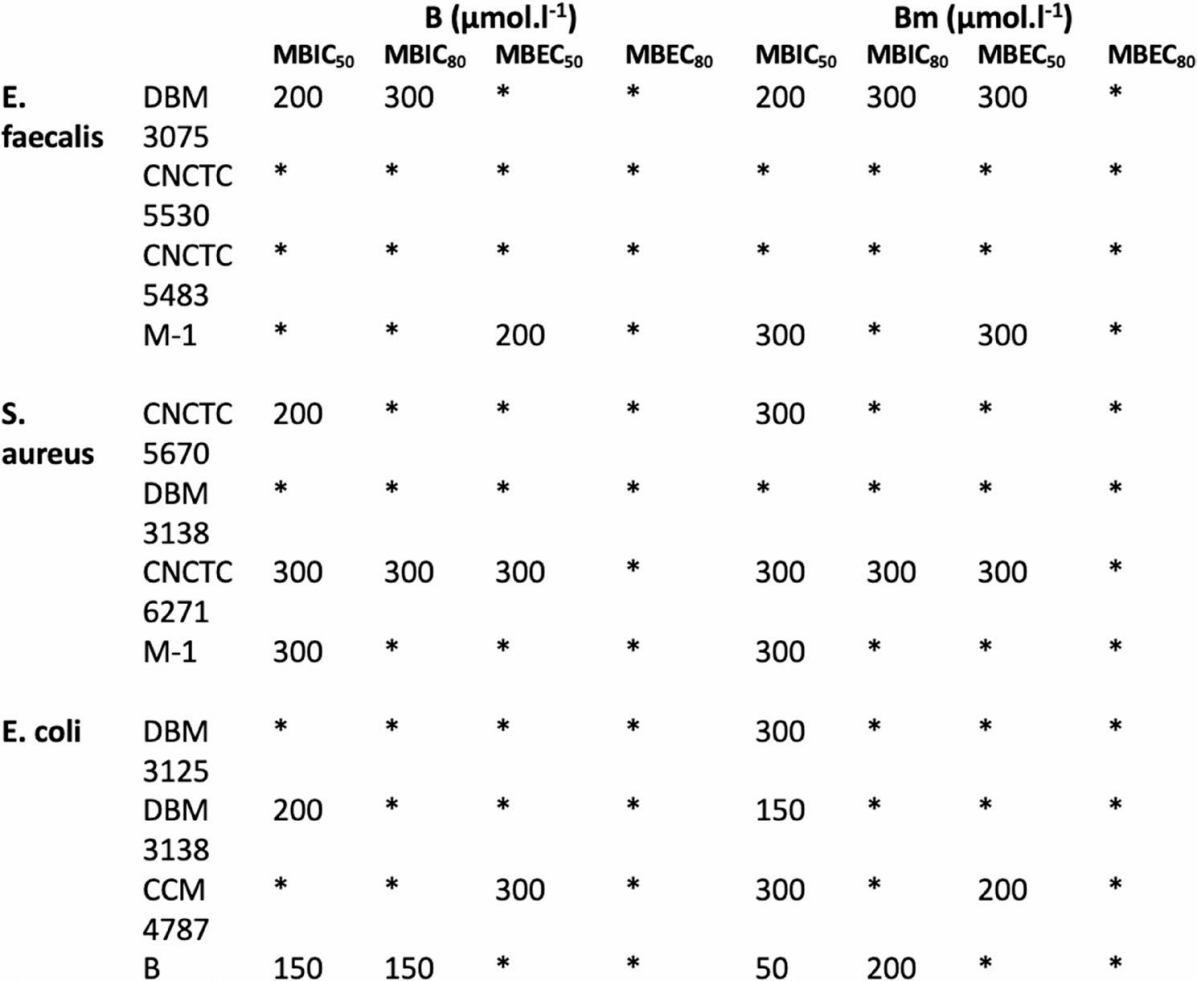
AMPs antimicrobial activity analysis. The effect of B and Bm peptides on biofilm formation and eradication of mature biofilm of *Enterococcus faecalis*,* Staphylococcus aureus* and *Escherichia coli* referred as MBIC50, MBIC80, MBEC50 a MBEC80. * Not detected in the concentration range (50–300 µM)

The predicted AMPs were measured for their potential antimicrobial activity in solution and against bacterial biofilms, given that dental plaque primarily consists of bacterial biofilms. For this purpose, three types of bacteria were selected: *Enterococcus faecalis* and *Staphylococcus aureus*, which are commonly associated with dental caries, and *Escherichia coli*, a bacterium found in the oral cavity, contributing to the diversity of bacterial species under the investigation [39, 41]. The identified AMBN derived peptide sequences demonstrated the ability to prevent both the formation of biofilms and also the eradication of biofilms. Additionally, the minimal inhibitory concentration (MIC_50_), minimal biofilm inhibition concentrations (MBIC_50_ and MBIC_80_) and minimal biofilm eradication concentrations (MBEC_50_ and MBEC_80_) were determined. Specifically, the peptides were dissolved in MQ water (pH = 7.4) and tested for their efficacy against both planktonic bacteria and biofilm-forming bacteria. The analyses were conducted using a resazurin viability assay. The tested A, Am, B and Bm peptides showed, that there is no significant activity referred as MIC_50_ towards suspension growth (Fig. 2), yet biofilm formation and development were inhibited (Figs. 3 and 4). Peptides A and Am were moderately effective in biofilm inhibition, as the MBIC_50_ values were obtained in studied range of concentrations for all strains of *E. faecalis*, *S. aureus* and *E. coli* including drug resistant clinical isolates. Peptides B and Bm were rather effective towards gram-positive specimen and interestingly, the greatest effect was observed in the case of methicillin-resistant strain of *S. aureus* CNCTC 6271, in which case both inhibition of biofilm attachment and even biofilm eradication was achieved (MBIC and MBEC values were found for 300 µM). Peptide Bm has mild inhibiting activity towards biofilm formation in all studied strains, yet eradication of biofilm was not achieved. The absence of planktonic inhibition by Peptide A, despite its marked antibiofilm efficacy, suggests that its mechanism may involve biofilm matrix disruption or inhibition of bacterial adhesion rather than direct bactericidal action. Similar activity profiles have been described for other AMPs that selectively target biofilm integrity without affecting planktonic growth. The findings indicate that the designed AMPs exhibited interesting antimicrobial properties against both Gram-positive and Gram-negative bacteria. Moreover, these AMPs have demonstrated the capability to inhibit the formation of new biofilms and to eradicate existing biofilms.

### Toxicity of antimicrobial peptides

**Fig. 5:**
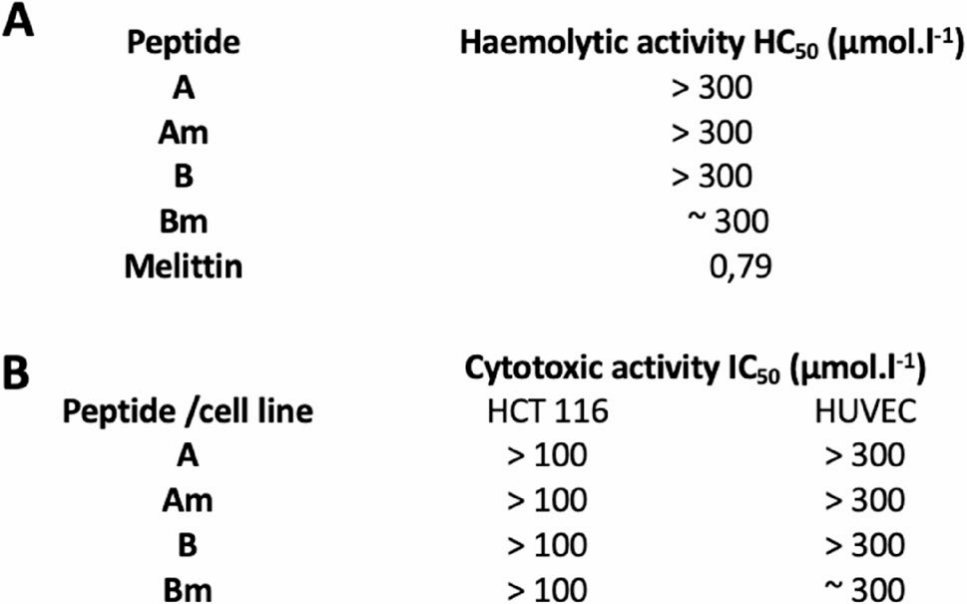
AMPs toxicity analysis. (**A**) Haemolytic activity of A, Am, B, Bm measured in the range of 12.5 to 100 (300) µM on a human blood sample and expressed as HC_50_. (**B**) Cytotoxic activity of A, Am, B, Bm peptides measured in the range of 12.5 to 100 (300) µM on HCT 116 and HUVEC cell lines, expressed as IC_50_

Fresh human whole blood collected from consenting healthy donors and treated with sodium citrate to prevent clotting, was used to assess the ability of the AMPs to lyse red blood cells. Melittin, a well-characterized amphipathic peptide from honey bee venom known for its potent haemolytic activity [55], served as a positive control. The haemolytic activity of the tested compounds was compared to that of 0.5% Triton X-100, which induced complete haemolysis. The concentration at which half of the red blood cells underwent lysis (haemolytic activity value, HC50) was used as an indicator of the peptide toxicity. The data presented in Fig. 5 A indicate that the HC50 values for peptides A, Am, B were significantly higher than 300 µM. The HC50 value of peptide Bm was determined to be approximately 300 µM. As anticipated, the positive control peptide Melittin exhibited a significantly lower HC50 of 0.79 µM.

The cytotoxicity of the tested AMPs was assessed on HUVEC (human endothelial cells) and HCT 116 (human colorectal carcinoma cells) cell lines. The concentration of the peptides required to achieve 50% inhibition of cell viability, known as IC50, was determined. The IC50 values, indicating the cytotoxic activity of the peptides, are presented in Fig. 5B. The IC50 values for the cytotoxic effect of the tested AMPs on HCT 116 cell lines were significantly greater than 100 µM. For HUVEC cells, the IC50 values were much higher than 300 µM for peptides A and B. Additionally, IC50 values closer to 300 µM were observed for peptides B and Bm.

### Antimicrobial peptides coated on titanium surface

**Fig. 6:**
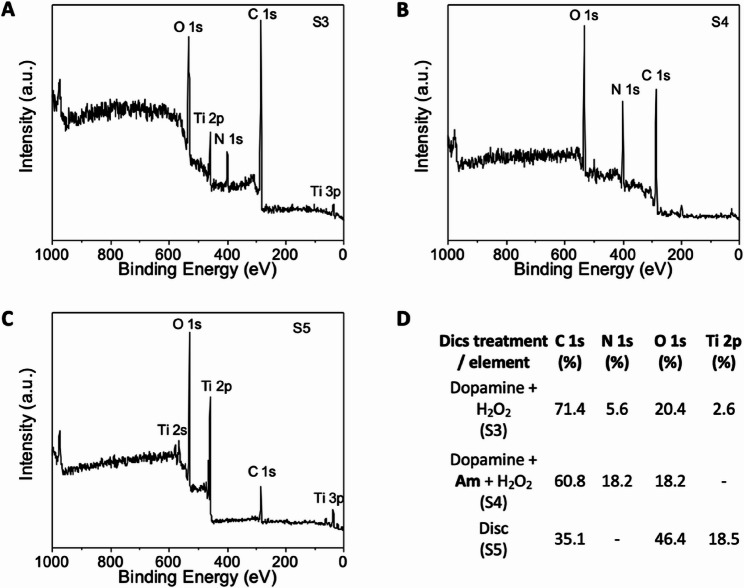
XPS analysis of Am peptide coated on TiO_2_ disc. The analysis was performed on three types of surfaces. The panel (**A**) represents TiO_2_ disc with dopamine and peroxide on TiO_2_ surface (S3); (**B**) represents dopamine, peroxide and Am peptide coated on TiO_2_ surface (S4, see more details about AMPs coating on TiO_2_ discs in Methods, Sect. 2.12.); (**C**) represents negative control as clean TiO_2_ disc (S5). (**D**) Table describes S3, S4 and S5 surfaces with analysis of C, N, O and Ti presence. S4 disc analysis indicated presence of N on TiO_2_ surface corresponding to presence of Am peptide. The negative controls revealed significantly lower (S3 - dopamine) or no presence (S5) of N on the disc surface

The process of coating AMPs onto TiO_2_ discs involved several strategic steps to ensure effective immobilization. Initially, the TiO_2_ discs were prepared with a clean surface using chemical etching and ultrasonication to remove contaminants and enhance surface roughness, which can improve peptide adhesion. Then dopamine layer was absorbed on the TiO_2_ surface with the following peptide coating. XPS method was chosen as a representative technique that provides information about the effectiveness of the surface modification. Atomic composition of dopamine and peptide coated TiO_2_ discs surface is shown in the Fig. 6. The XPS survey results demonstrated that the dopamine was transferred to the TiO_2_ surface. Narrow scan spectra and fitting curves compare the TiO_2_ disc coated with dopamine (S3), TiO_2_ disc coated with dopamine and Am peptide (S4), and clear TiO_2_ disc (S5) providing a negative control. For dopamine (S3) and dopamine with Am (S4) discs, an increase of the atomic percentage of carbon and nitrogen can be observed. Both films are characterized by decreasing the percentage of oxygen. The content of nitrogen as a typical indicator of protein content showed significant increase at the Am coated TiO_2_ disc (S4) and confirmed the presence of the peptide on the disc surface. The nitrogen presence, although typical for dopamine (S3), was significantly lower than for the Am-treated disc, and no nitrogen was detected on the clear TiO_2_ disc (S5). The XPS results confirmed stable presence of Am peptide on TiO_2_-dopamine layered disc suggesting plausible utilization of the material for potential antimicrobial medical treatment.

QCM-D is a surface sensitive technique that measures the changes in the oscillation frequency of a quartz crystal sandwiched between two gold electrodes. Since the frequency changes (ΔF) can be associated with mass deposited onto/released from the surface [56, 57], this method can be used to study adsorption/desorption phenomena on biomolecules, such as peptides. Additionally, the setup used in this study can also measure the dissipation (ΔD), related to the energy loss produced by the deposited film [58–60]. The QCM-D data clearly showed that A and Am peptides bonded irreversibly to the titanium sensors (Figure S1). Within the first minute of adsorption, both frequency and dissipation change very fast, almost vertically. This behaviour is indicative for a very fast adsorption. Then, as the binding slows down, the slopes gradually become horizontal. This suggests that the binding sites are less abundant, and thus the adsorption process slows down [60]. The method confirmed stability of AMPs coating on TiO_2_ surface.

## Discussion

This study combined proteolytic profiling of human AMBN ISO I with *in silico* prediction tools to identify and design four candidate antimicrobial peptides. The approach aligns with recent strategies leveraging endogenous host proteins as AMP precursors, where protease-generated fragments often exhibit enhanced antimicrobial function compared with full-length proteins [7, 61–63]. The identification of two naturally occurring AMBN fragments and two engineered derivatives is consistent with previous observations that enamel matrix proteins can release bioactive peptides with antimicrobial properties [5, 6]. Our findings therefore broaden the emerging understanding that enamel matrix proteins may contribute not only to biomineralization but also to innate antimicrobial defence within the oral cavity.

The activity profiles of the AMBN-derived peptides were dominated by antibiofilm rather than planktonic inhibition, which agrees with the known behaviour of many AMPs whose primary mechanism targets sessile bacterial communities [9–11]. Biofilms display enhanced tolerance to antibiotics, and AMPs have been shown to disrupt them through multiple pathways, including membrane permeabilization, suppression of quorum sensing, or interference with extracellular polymeric substances [15, 32, 64].

In the study, peptides A and Am demonstrated moderate inhibition across Gram-positive and Gram-negative species, whereas peptides B and Bm were more selective toward Gram-positive bacteria, with B exhibiting notable activity against methicillin-resistant S. aureus CNCTC 6271. This pattern is consistent with structural determinants observed in other α-helical AMPs, where increased hydrophobicity or charge distribution correlates with activity against thick peptidoglycan layers [64]. Secondary structure analysis further supports this interpretation. CD spectroscopy confirmed that all peptides were largely disordered in aqueous solution but gained α-helical content in the presence of TFE, a structural mimic of membrane-like environments (Figure S1). Many cationic AMPs are known to adopt amphipathic α-helices upon membrane contact, enabling barrel-stave or toroidal pore formation [65]. Confocal microscopy using fluorescently labelled Am peptide demonstrated membrane surface coverage and partial penetration of *E. coli* cells (Figure S2), consistent with pore-forming or membrane-disruptive mechanisms [28, 66]. While our data support a membrane-targeting model, it is likely that additional biofilm-specific mechanisms contribute to the observed effects, such as inhibition of attachment or metabolic suppression, as AMPs often exhibit multifunctional antibiofilm pathways [26, 30, 64]. Additionally, for example lactose dehydrogenase (LDH) release assay could also provide further insight into membrane integrity, although current data already indicate excellent cytocompatibility of the peptides. Future work should include LDH-based analyses to validate these observations. Further mechanistic studies will be required to delineate these contributions.

An essential advantage of AMPs for clinical translation is their generally favourable safety profile. In this study, all peptides exhibited low cytotoxicity toward mammalian cells and minimal haemolytic activity, in agreement with widely reported AMP characteristics [64]. The high IC₅₀ and HC₅₀ values observed for A, Am, B, and Bm support their suitability for biomedical application (Fig. 5). Additionally, AMPs typically demonstrate low rates of resistance development due to their rapid and physical modes of action on membranes [28, 67]. Their potential synergistic activity with conventional antibiotics [68] further enhances their relevance for managing infections where standard therapies fail, such as persistent biofilms in periodontal disease, peri-implantitis, or carious lesion progression [31, 69–72].

The successful immobilization of the peptides on TiO₂ surfaces represents a key translational advance. Titanium remains a preferred material for oral and orthopaedic implants due to its biocompatibility and mechanical strength [36, 73, 74]. Coating titanium with AMPs offers a strategy for preventing early-stage bacterial adhesion, one of the primary causes of implant failure. Our XPS and QCM-D analyses confirmed that peptides—especially A and Am—formed stable, irreversible coatings. These results align with previous studies demonstrating that immobilized AMPs can maintain or enhance antimicrobial efficacy and benefit from increased stability compared with soluble forms [75, 76]. The possibility of combining AMP coatings with titanium’s inherent surface properties, including photoactivity, raises additional opportunities for synergistic antimicrobial strategies [77]. The XPS measurement accompanied with QCM-D technique confirmed stable coating of the peptides on the TiO_2_ surface. The QCM-D data showed that A and Am peptides bonded irreversibly to the titanium sensors (Figure S3). Quantitative determination of peptide surface loading will be addressed in future work using fluorescence labeling or spectroscopic methods to better correlate surface density with antimicrobial efficacy. The combination of TiO_2_’s inherent biocompatibility and the antimicrobial efficacy of the identified AMPs may provide a synergistic approach to combatting microbial colonization on biomedical surfaces. In order to confirm preserved or even improved antimicrobial properties of the coated AMPs on TiO_2_ discs further research would be required.

The limitations of this study represent in vitro environment of performed experiments, which does not fully recapitulate the dynamic and polymicrobial environment of oral biofilms. Biofilm architecture, salivary proteins, and host immune factors may influence AMP performance differently in vivo. Moreover, while our coating methods produced stable layers, the long-term durability, mechanical stability, and antimicrobial efficacy of AMP-coated implants require validation under physiologic conditions. This study focused on the intrinsic antimicrobial and antibiofilm potential of AMBN-derived peptides. Future investigations should include clinically relevant antibiotics (e.g., ampicillin, chlorhexidine, gentamicin) as benchmarks to facilitate direct comparative evaluation of peptide efficacy. Future studies should also include animal models, assessment of peptide release kinetics, and evaluation of synergistic effects with existing antimicrobial agents or implant technologies.

## Conclusion

Peptides derived from AMBN ISO I exhibited significant antimicrobial and antibiofilm activity while maintaining low cytotoxicity. Their efficacy against biofilm-forming and antibiotic-resistant bacteria highlights their potential as novel antimicrobial agents. Importantly, the successful immobilization of these peptides on titanium surfaces demonstrates their applicability as bioactive coatings for oral implants. Such coatings could offer localized, sustained protection against biofilm-related infections, potentially improving implant longevity and reducing reliance on systemic antibiotics. Further studies are warranted to optimize coating stability, evaluate long-term biocompatibility, and confirm antimicrobial efficacy in vivo. Overall, this work expands the understanding of AMBN-derived peptides as multifunctional molecules with both developmental and antimicrobial roles. It demonstrates their capacity to inhibit clinically relevant biofilms and highlights their potential for integration into next-generation antimicrobial coatings for dental biomaterials—addressing a critical need in preventing implant-associated infections.

## Supplementary Information

Supplementary Material 1.

## Data Availability

The datasets used and/or analysed during the current study are available from the corresponding author on reasonable request.

## Abbreviations

ACPs: Anti Cancer Peptides
AMP: Antimicrobial Peptides
APD: Antimicrobial Peptides Database
CD: Circular dichroism
ECD: Electronic circular dichroism
EDTA: Ethylene diamine tetra acid
FBS: Fetal serum albumin
HBTU: O-(Benzotriazol-1-yl)-N,N,N',N'-tetramethyluronium hexafluorophosphate
HC50: Concentration at which half of red blood cells are lysed (hemolysis)
HUVEC: Human Umbilical Vein Endothelial Cells
IC50: inhibitory concentration at 50% cytotoxicity
IDP: Intrinsically Disordered Protein
MBIC50: Minimum concentration for 50% biofilm inhibition
MBIC80: minimum concentration for 80% biofilm inhibition
MBEC50: Minimum concentration for 50% biofilm eradication
MIC50: minimum inhibitory concentration to 50% reduction in the increase of suspension cells
MMP: metalloproteases
N-Fmoc: Fluorenylmethyloxycarbonyl (protecting group for organic synthesis)
PBS: Phosphate buffer
PMSF: Phenylmethylsulfonyl fluoride
SDS: Sodium dodecyl sulfate
SDS-PAGE: Electrophoresis in a polyacrylamide gel
TEV: Tobacco a etch virus
TFE: Trifluoroethanol
RP-HPLC: High-performance liquid chromatography on a non-polar sorbent
RT: Room temperature
EDTA: Ethylenediamine terephthalic acid
MQ: Ultrafiltered water from Merck
