# Supplementary material for: Antimicrobial peptides derived from human ameloblastin targeting biofilms
Source: BMC Oral Health. 2025 Dec 2;26:24. doi: 10.1186/s12903-025-07433-w (PMC12777444; doi:10.1186/s12903-025-07433-w)
Supplement: Supplementary file 1 — Supplementary Material 1. [file 12903_2025_7433_MOESM1_ESM.docx]

**Supplementary material**

Antimicrobial Peptides from Human Ameloblastin Targeting Biofilms

Veronika Vetyskova ^1,^ **, Petra Kasparova ^2,^ **, Lucie Bednarova ^1^, Miroslav Hajek ^1^, Jan Luxa ^3^, Alejandro Barrantes Bautista ^4^, Jane Elin Reseland ^5^, Olga Matatkova ^2^, Jan Masak ^2^, Jiri Vondrasek ^1,^ * and Kristyna Vydra Bousova ^1,^ *

1. Institute of Organic Chemistry and Biochemistry of the Czech Academy of Sciences, Flemingovo namesti 2, 16000 Prague, Czechia.
2. Department of Biotechnology, University of Chemistry and Technology Prague, Technicka 5, 166 28 Prague, Czechia.
3. University of Chemistry and Technology, Prague, Technická 5, Prague 6, 166

28, Czechia.

^4^  Oral Research Laboratory, Institute for Clinical Dentistry, University of Oslo, Norway

^5^ Department of Biomaterials, Institute of Clinical Dentistry, University of Oslo, Oslo, Norway

** Authors contributed equally

***** Correspondence: [kristyna.bousova@uochb.cas.cz](mailto:kristyna.bousova@uochb.cas.cz); [jiri.vondrasek@uochb.cas.cz](mailto:jiri.vondrasek@uochb.cas.cz), Tel.:420-220-183-131


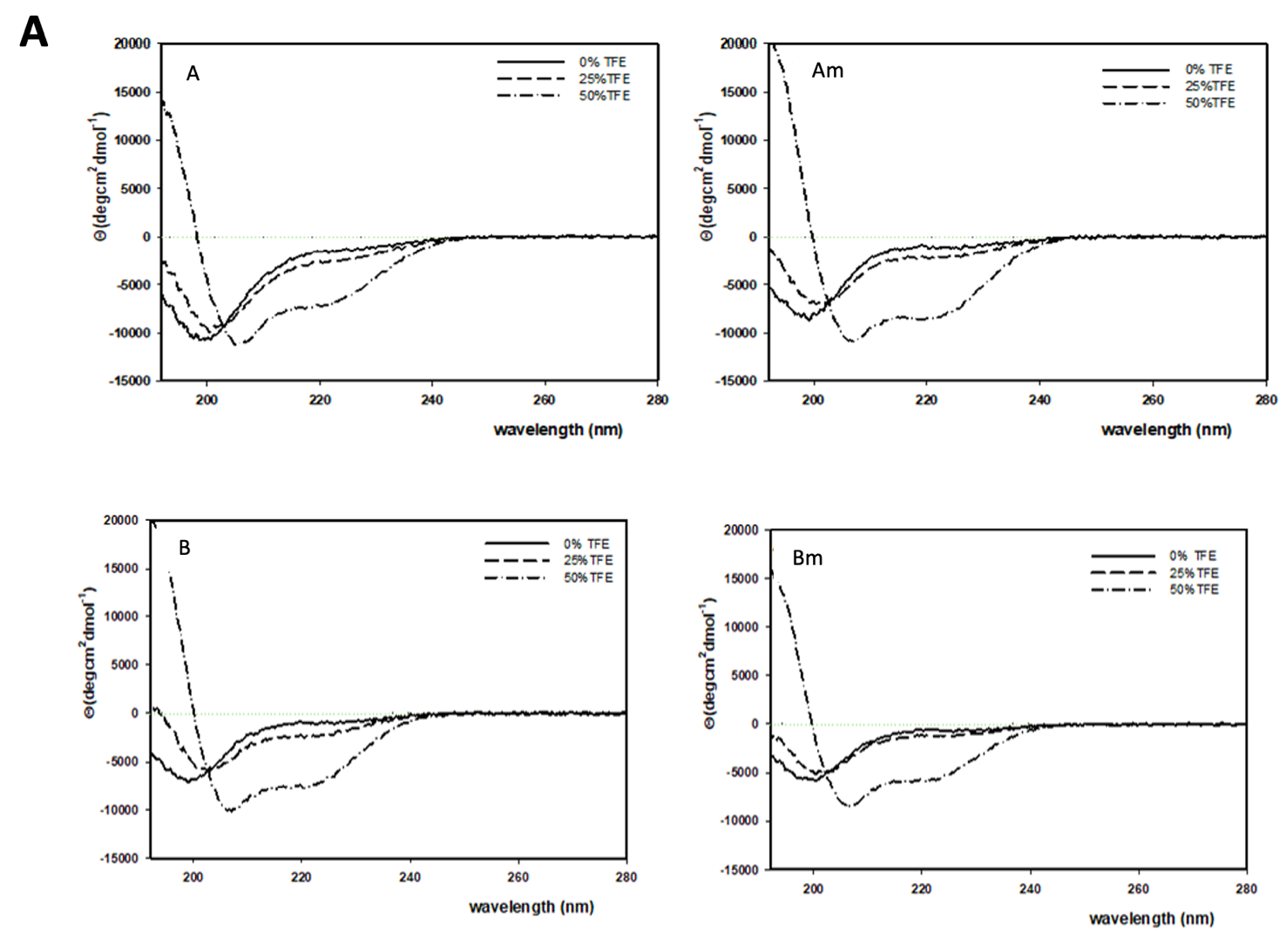


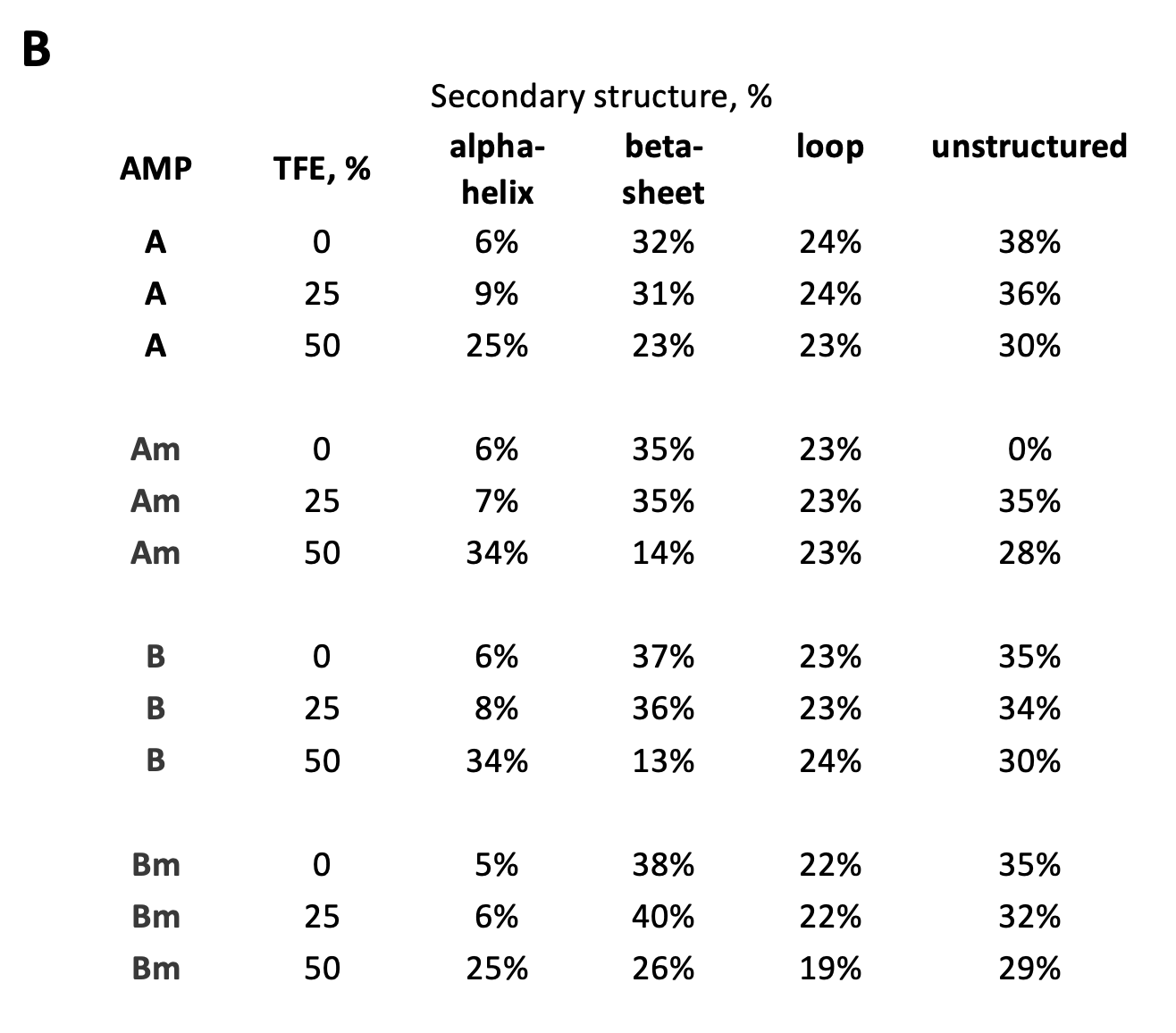


**S1 Circular dichroism spectra of AMPs.**  **(A)** CD of A, Am, B, Bm peptides in the presence of 0%, 10%, 30% and 50% (v/v) TFE. **(B)** Table of secondary structural analysis by CD technique provides a summary of the proportions of secondary structures and their propensity to adopt helical structures. Calculated incidence (%) of secondary structural content determined by CD spectroscopy.


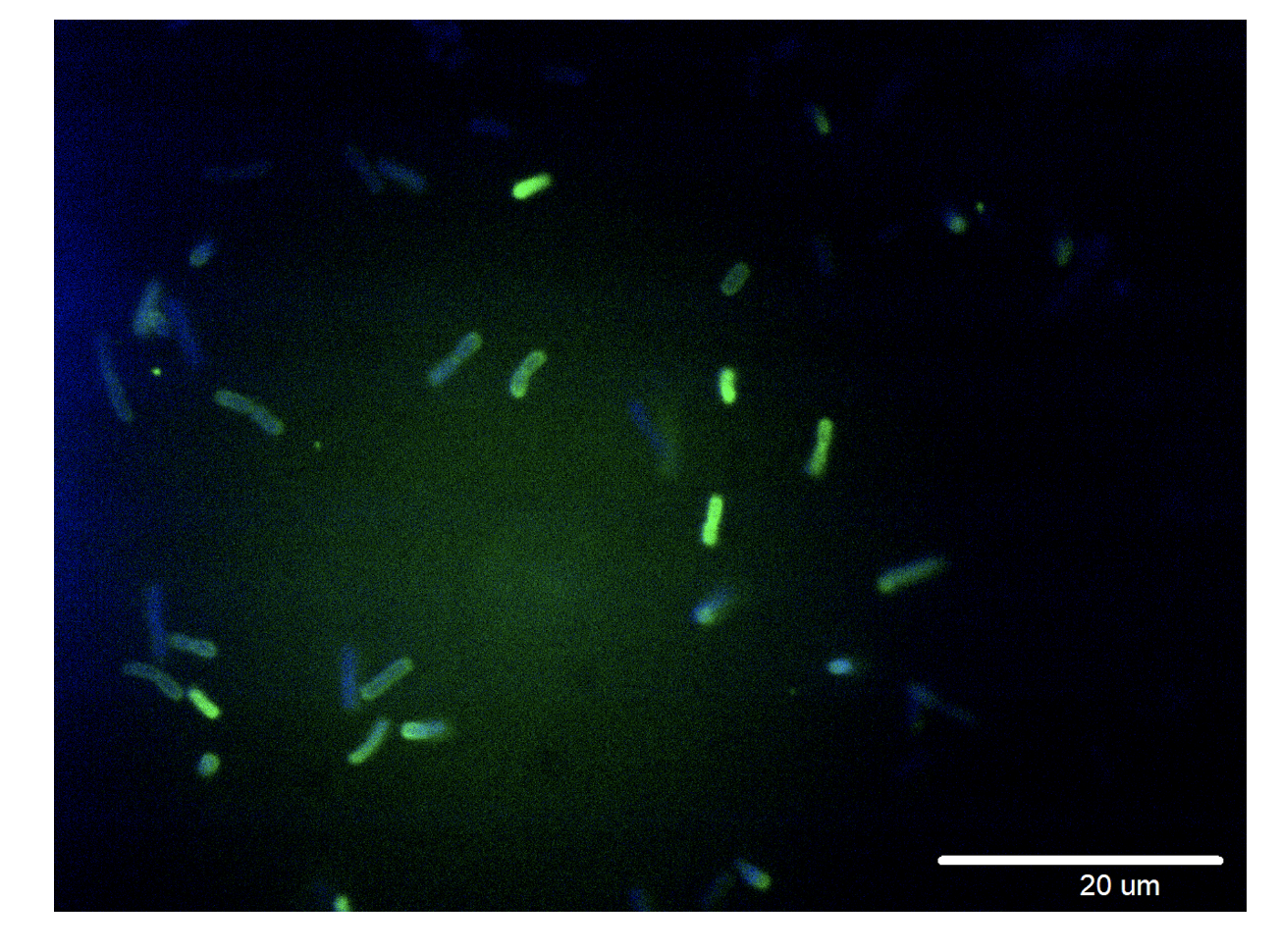
 **S2** Confocal micrographs depicting localization of A-peptide fluorescently labelled peptide (green signal) by double-staining of adhered cells of *Escherichia Coli* with DAPI (blue signal). Adhered cells double-stained by DAPI and Fl-LL-III/43 showing A peptide attached to the cell membrane.

**
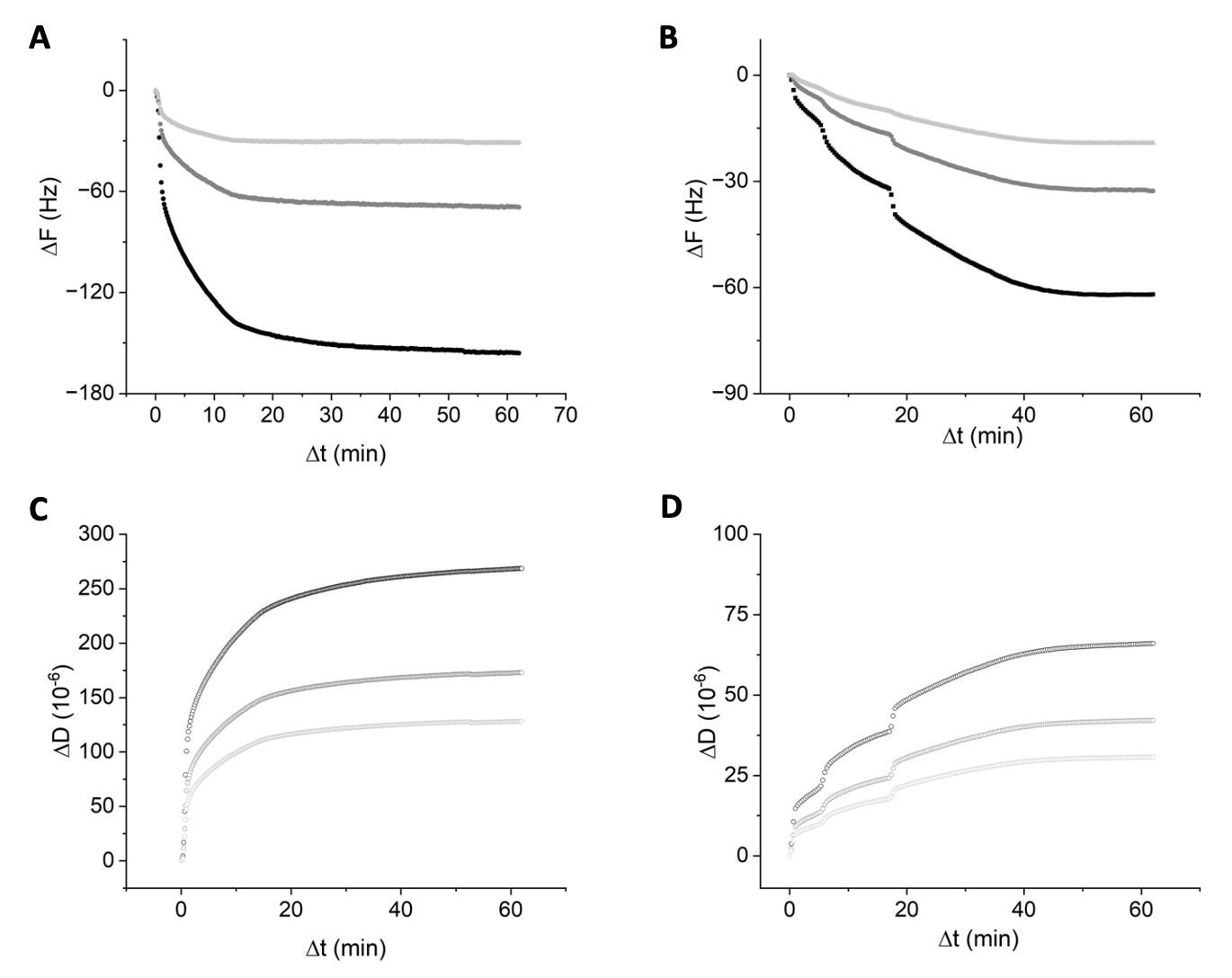
**

**S3** QCM-D results showing the adsorption of both (A, C) A- and (B, D) Am- peptides onto titanium surfaces. Measurements were carried out at 21 ºC in 10mM Tris supplemented with 100 mM NaCl at pH 7.5.
